# Intracranial venous pressures and endovascular outcomes in pediatric patients with cerebral venous sinus stenosis

**DOI:** 10.1007/s00381-025-06938-7

**Published:** 2025-09-09

**Authors:** Sage P. Rahm, Matthew Thomas Cleveland Jarrell, Sasha Howell, Anil Mahavadi, Anastasia Smith, James M. Johnston, Jesse G. A. Jones

**Affiliations:** Department of Neurosurgery, Division of Pediatric Neurosurgery, University of Alabama at Birmingham, Children’s of Alabama, 1600 7TH Avenue South, Lowder 400, Birmingham, AL 35233 USA

**Keywords:** Cerebral venous sinus stenosis, Venous sinus pressures, Idiopathic intracranial hypertension, Venous sinus stenting, Balloon angioplasty, Diagnostic cerebral venogram

## Abstract

**Purpose:**

Diagnostic cerebral venograms are the gold standard for evaluating cerebral venous sinus stenosis (CVSS). Venous sinus stenting (VSS) and less commonly venous sinus angioplasty are emerging endovascular treatments in pediatric patients. This study examines the baseline intracranial venous pressures and postoperative endovascular outcomes in pediatric patients with CVSS.

**Methods:**

A retrospective chart review was performed on patients ≤ 18 years old with CVSS between October 2021 and August 2024.

**Results:**

A total of fifteen patients with CVSS underwent 20 endovascular procedures. The average age was 13.0 years of age (IQR 6–15 years of age) and 53.3% female. Eight patients (53%) were diagnosed with IIH by the revised Friedman criteria. Papilledema was present in 73.3% of patients with an average lumbar puncture or ventriculostomy opening pressure of 409 mmH_2_O. The average superior sagittal sinus (SSS) pressure was 24.3 mmHg (range 13–50 mmHg). The average trans-stenotic gradient was 8.5 mmHg (IQR 4.0–13.3; n = 14). Five patients underwent dural venous sinus stenting (mean pre-stent TSG of 17.0) with a significant reduction in the trans-stenotic gradient of 13.5 mmHg (p = 0.04; 79.4% relative reduction). One of these VSS patients developed stent adjacent stenosis (SAS) at follow-up requiring further venous sinus stenting. There was one peri-operative complication (5%) involving a retroperitoneal hematoma.

**Conclusion:**

Initial dural venous sinus stenting significantly reduced the trans-stenotic gradient in all pediatric CVSS patients. Of the patients who underwent venous sinus angioplasty, 100% required further surgical intervention for management of their ICPs. There was one perioperative complication (5%) associated with CVSS endovascular interventions.

## Introduction

Cerebral venous sinus stenosis (CVSS) is a rare condition and is frequently associated with idiopathic intracranial hypertension (IIH) [1]. Pediatric IIH has an incidence of less than 1 per 100,000 children [2, 3]. Although rare, elevated intracranial pressures may significantly affect a child’s development by causing headaches, papilledema, vision loss, diplopia, or tinnitus due to elevated intracranial pressures [4–6]. Early recognition is crucial, as untreated intracranial hypertension in a child may potentially result in permanent visual impairment or other neurologic sequelae. ​

Diagnostic cerebral venograms are the gold standard for evaluating the degree of CVSS and intracranial venous pressures. Venous sinus stenting (VSS), and less commonly venous sinus angioplasty, can be utilized to restore the caliber of a stenosed sinus and improve cerebral venous outflow. Generally, a pressure gradient (commonly ≥ 8 mmHg across the stenosis) is used as one of the criteria to justify intervention ​[7]. However, these thresholds are based on adult data and no clear pressure gradient cutoff has been established for children​.

Early pediatric case reports and small series have suggested that VSS can be performed safely in older children and adolescents with CVSS with low peri-procedural morbidity comparable to adults​ [7, 8]. However, the long-term efficacy and safety of venous angiograms for diagnosis and venous sinus stenting and venous sinus angioplasty for treatment in children remain uncertain. This study examines the baseline intracranial venous pressures, postoperative endovascular complication rates, and outcomes of venous sinus stenting and venous sinus angioplasty in pediatric patients with CVSS.

## Methods

Following Institutional Review Board (IRB) approval, we conducted a retrospective chart review of pediatric patients diagnosed with CVSS from October 2021 through August 2024 at our institution. Patients were included who had a confirmed CVSS diagnosis based on diagnostic cerebral venograms and who were ≤ 18 years old at the time of diagnosis. Patients without diagnostic venograms were excluded.

Demographic variables abstracted from the electronic record included age at first procedure, sex, race or ethnicity, height and weight. Body-mass index (BMI) was calculated as weight/height^2^ (kg m⁻^2^) and converted to a CDC age-adjusted percentile for patients two years of age or older. Presenting features recorded included headache, papilledema, visual disturbance, cranial-nerve palsy, tinnitus, vertigo, seizures, emesis, heart failure, and stroke. We documented use and dose of acetazolamide at presentation and at last follow-up. Opening pressure was taken from the first lumbar puncture or, when available, intraparenchymal or ventricular monitoring.

IIH was diagnosed utilizing the revised Friedman criteria: an opening pressure ≥ 18 mmHg (250 mmH_2_O) unsedated, non-obese children or ≥ 20 mmHg (280 mmH_2_O) in sedated or obese children, presence of papilledema, no evidence of hydrocephalus on radiological imaging, normal cerebrospinal-fluid (CSF) composition, and no neurological deficit other than a cranial-nerve palsy. All other cases were classified as secondary intracranial hypertension if applicable.

For every venographic session we recorded the sinus involved (transverse, sigmoid, superior sagittal, torcula or transverse–sigmoid junction). Superior sagittal-sinus pressure and the trans-stenotic gradient (proximal minus distal venous pressure) were documented before, and when applicable after, an intervention. Technical variables captured were vascular access site, arterial and venous sheath size, contrast volume, fluoroscopy time, cumulative dose-area product measured in milligray (mGy), and total procedure duration.

Procedures were categorized as diagnostic venography, diagnostic cerebral angiogram, balloon angioplasty, VSS placement, or combined therapy. Post-operative complications were logged, including vascular injury, hemorrhage, stroke, or death. Clinical outcome at each follow-up visit was graded as resolved, improved, unchanged or worsened for headache, papilledema, and visual complaints. Acetazolamide dose changes, new ventriculoperitoneal shunt placement and any repeat endovascular procedure were recorded. Follow-up time was calculated from the initial venographic session to the most recent clinic or telehealth encounter.

Continuous data were assessed for normality with the Shapiro–Wilk test. Variables with a normal distribution are presented as mean ± standard deviation, whereas non-normal variables are given as median and inter-quartile range. Categorical variables are expressed as counts and percentages. We compared paired pre- and post-intervention values with the Wilcoxon signed-rank test. Effect size was reported as the Hodges–Lehmann median paired change with its exact 95% CI and the matched-pairs correlation (*r*). A two-sided *p*-value < 0.05 was considered statistically significant. Statistical analyses were conducted with SPSS Statistics, version 28 (IBM Corp., Armonk, NY, USA).

## Results

### Demographics

A total of 15 pediatric patients with CVSS who underwent diagnostic cerebral venograms were included in this study, accounting for 20 total endovascular procedures. Median age at first endovascular procedure was 13.0 years (IQR 6–15). 53.3% of patients were female and 93.3% were white (Table 1). The mean baseline BMI was 24.8 ± 7.9 kg m⁻^2^ (n = 14), corresponding to a median age-adjusted percentile of 87.7% (IQR 53.4–97.4, n = 14) (Table 1). Eight patients (53.3%) were diagnosed with IIH, while 7 patients did not meet the revised Friedman criteria. Mean baseline CSF opening pressure was 409 mmH₂O (Table 1). The most common presentations were papilledema (73.3%), headache (73.3%), and vision changes (40%) (Table 1).

**Table 1:** Baseline patient characteristics

Patient	Age (Years)	Sex	BMI	BMI Age Percentile	Underlying Condition	Initial CSF Opening Pressure (mmH_2_O)	Headache	Papilledema
1	15	F	37.8	99	CSF opening pressure < 280 mmH_2_O and obese, does not meet revised Friedman criteria	250	-	+
2	11	M	25	98	CSF opening pressure < 280 mmH_2_O and obese, does not meet revised Friedman criteria	244	-	+
3	13	F	23.6	85	IIH	260	-	+
4	15	F	22.3	54	No Papilledema, does not meet revised Friedman criteria	370	+	-
5	13	F	16.9	24	IIH	480	+	+
6	15	F	25.7	91	IIH	367	+	+
7	6	M	15.8	63	IIH	510	+	+
8	6	M	22.9	68	Secondary IH -Hydrocephalus	680	+	-
9	10	M	23.7	96	IIH	480	-	+
10	15	M	42.1	100	Secondary IH—Inflammatory/Infectious	370	+	+
11	4	F	13.6	7	IIH	360	+	+
12	15	F	31	97	IIH	550	+	+
13	17	M	26.8	90	Secondary IH—Hydrocephalus	476	+	-
14	1	M	N/A	N/A	IIH	335	+	+
15	15	F	20.3	52	No Papilledema, does not meet revised Friedman criteria	UNK^a^	+	-

a= Diagnosed from the diagnosis in outside hospital note which is IIH. No CSF studies

### Procedures

Among the twenty procedures, six included venous sinus stent placement, four included balloon angioplasty, and ten were purely diagnostic which all included venography (Table 2). Mean fluoroscopy time for all sessions was 22.6 ± 11.6 min, the mean air-kerma was 321.3 ± 231.0 mGy, the mean contrast volume was 32.0 ± 16.6 mL, and the mean procedure time was 101.5 ± 31.5 min.

**Table 2:** Endovascular procedure details

Patient	Number of Prior Endovascular Procedures	Age at Time of Procedure (years)	Procedure Type	Contrast (mL)	Fluoro (min)	Air-kerma (mGy)^a^	Procedure Time (min)	Peri-operative Complication
1	0	15	DV	17.6	11.2	126	87	0
	1	15	DV, VS	30.6	24.2	326	111	0
2	0	11	DCA, DV	40.6	17.2	197	101	0
3	0	13	DCA, DV	23	13.1	125	91	0
4	0	15	DV	14	10.1	83	56	0
5	0	13	DCA, DV	22.5	9.9	62.5	57	0
6	0	15	DCA, DV	10	UNK	330.17	103	0
	1	15	DV, VS	10	UNK	316.86	72	0
	2	17	DCA, DV	30	9.1	135	83	0
	3	17	DV, VS, BA	32	27.1	497	138	0
7	0	6	DV, VS	24	15.1	172	104	0
8	0	6	DCA, DV, BA	38	21.8	191	145	0
9	0	10	DCA, DV	51.6	18	321	125	0
	1	10	DCA, DV, VS	41.6	36.7	752	168	0
10	0	15	DCA, DV	40.7	14.5	219	107	0
11	0	4	DCA, DV, BA	40	50.5	429.97	125	0
12	1	15	DCA, DV, VS	41	30.1	733.07	86	Retroperitoneal hemorrhage
13	0	17	DCA, DV	75	33	877.65	113	0
14	1	1	DV, BA	8	34.5	264.56	40	0
15	0	15	DCA, DV	49	31.5	266.6	118	0

*DV* = diagnostic cerebral venography, *VS* = venous sinus stenting, *DCA* = diagnostic cerebral angiogram, *BA* = venous sinus balloon angioplasty

a= Some procedures were performed on dated angiography equipment, which increased radiation dose compared to those performed on a newly installed biplane unit

### Hemodynamic effect

On baseline venogram, 8 patients had stenosis at the transverse/sigmoid junction, 4 had stenosis in the transverse sinus, 2 at the sigmoid sinus, and 1 patient had stenosis at the sigmoid/internal jugular vein junction as well as the superior sagittal sinus. Of these initial venogram findings, stenosis was on the right side in eight patients, on the left in four, and bilateral in two (Table 3).

**Table 3:** Baseline radiologic and hemodynamic findings

Patient	Stenosis Side	Stenosis Location	Right Pressure Gradient (mmHg)	Left Pressure Gradient (mmHg)
1	Right	TS J	8	N/A
2	Right	TS	7	N/A
3	Right	TS J	7	N/A
4	Right	TS	4	N/A
5	Bilateral	TS	1.5	2.2
6	Left	TS J	N/A	9
7	Right	TS	17.7	N/A
8	Right	TS J	10	N/A
9	Right	SS	11.8	N/A
10	Left	TS J	N/A	UNK
11	Right	SI J & SSS	3	N/A
12	Right	TS J	22	N/A
13	Bilateral	TS J	29	35
14	Left	SS	N/A	9
15	Left	TS J	N/A	2

*TS J* = Transverse/Sigmoid Junction, *TS* = Transverse Sinus, *SS* = Sigmoid Sinus, *SI J* = Sigmoid/IJV Junction, *SSS* = Superior Sagittal Sinus

Baseline trans-stenotic gradient (TSG) was documented in 14 of the patients, yielding a median value of 8.5 mmHg (IQR 4.0–13.3) (if patient had bilateral stenosis the side with the largest gradient was used for calculation). Of the five first time VSS procedures, the gradient fell 13.5 mmHg (p = 0.04) from a mean of 17.0 ± 5.9 mmHg to 3.5 ± 3.3 mmHg, corresponding to a 79.4% relative reduction (Table 4). Furthermore, four of five stented children with a TSG > 8 achieved a post-stent TSG below 8 mmHg. One patient underwent a second VSS procedure due to stent adjacent stenosis with a TSG of 6 mmHg—after stenting the gradient remained unchanged at 6 mmHg. No patients in the series required bilateral VSS.

**Table 4:** Dural venous sinus stenting and balloon angioplasty procedure details

Patient	Age	Intervention Type	Prior Interventions	Stenosis Side	Stent (mm)	Same Session as Diagnostic Study	Pre-TSG (mmHg)	Post-TSG (mmHg)	TSG Decrease (mmHg)^*^
1	15	VS	None	Right	6 × 40	No	11	2	9
6	15	VS	None	Left	6 × 40	No	11	8	3
	17	VS	VS	Left	6 × 40	No	6	6	0
7	6	VS	None	Right	6 × 40	Yes	17.7	5.9	11.8
9	10	VS	None	Right	6 × 40	No	23.5	1.5	22
12	15	VS	None	Right	6 × 30	No	22	0	22
8	6	BA	Shunt	Right	N/A	Yes	10	UNK	UNK
11	4	BA	None	Right	N/A	Yes	3	UNK	UNK
14	1	BA	BA	Left	N/A	No	9	8	1

*VS* = venous sinus stenting, *BA* = venous sinus balloon angioplasty

*= Average reduction in trans-stenotic gradient for the first time VSS group was 13.5 mmHg (p = 0.04; 79.4% relative reduction)

### Clinical outcomes

Median follow-up for all fifteen children was 6 months (IQR 2–25); stented patients had a median follow-up of 6 months (IQR 3.5–29) whereas non-stented patients had a median follow-up of 7 months (IQR 0–26.3) (Table 5). Of the three stented patients who had headaches prior to stenting, headaches resolved or improved in all three (100%), compared with four of eight patients in non-stented children (50%). All five stented patients had papilledema, however it resolved or improved in three children (60%), whereas three of the four non-stented patients saw resolved or improved papilledema (Table 6). Lastly, the two children in the stent group with vision changes saw resolution after stenting (100%), whereas both children in the non-stented group with vision changes saw stabilization or improvement in their vision (100%). No stented child required ventriculoperitoneal shunting, whereas three in the non-stented group required permanent CSF diversion (30%).

**Table 5:** Clinical outcomes by treatment group

Treatment Group	Patients (n)	Median FU (mo) [IQR]	Mean Baseline Opening Pressure (SD)	Headache Improved	Papilledema Improved	Vision Stabilized/Improved	Needed VPS	Re-intervention
Stent	5	6 [3.5–29]	431.4 (122.1)	3/3 (100%)	3/5 (60%)	2/2 (100%)	0/5 (0%)	1/5 (20%)
No-Stent^a^	10	7 [0–26.3]	397.2 (133.3)	4/8 (50%)	3/4 (75%)	2/2 (100%)	3/10 (30%)	0/10 (0%)

a = Includes patients who had angioplasty alone

**Table 6:** Symptom improvement post-intervention

Stent^a^	Headache		Papilledema		Vision Changes		Acetazolamide	
Patient	Pre-Intervention	Post-Intervention	Pre-Intervention	Post-Intervention	Pre-Intervention	Post-Intervention	Pre-Intervention	Post-Intervention*
1	-	-	+	UNK	-	-	500 mg	500 mg
6	+	Improved	+	Improved	+	Improved	1500 mg	750 mg
6	+	Improved	+	Improved	+	Improved	1000 mg	1000 mg
7	+	Resolved	+	Improved	+	Resolved	350 mg	600 mg
9	-	-	+	Improved	-	-	1000 mg	500 mg
12	+	Resolved	+	UNK	-	-	3000 mg	None
Angioplasty								
8	+	Improved	-	-	-	-	None	None
11	+	No Change	+	Improved	-	-	None	10 mL
14	+	Improved	+	No Change	-	-	None	None

a= Blue: Patients with IIH

*= The median paired reduction in acetazolamide for the first time VSS group was −250 mg/day (p = 0.14)

### Subgroup analysis

IIH patients presented with a baseline mean TSG of 10.4 ± 6.6 mmHg compared to patients without IIH of 11.0 ± 12.1 mmHg. Four of eight patients with IIH received a stent (50%), while one of seven without IIH received a stent (14.3%). Regarding the rest of the IIH patients, an additional two underwent angioplasty alone (25%) and two were managed conservatively. Among the six IIH children who presented with headache, five (83.3%) reported resolution or clear improvement at last review; five of those six had undergone an endovascular intervention (VSS or angioplasty). Papilledema was present in all eight IIH children and improved or resolved in four (50%), with follow-up unavailable for three cases including two stented children. In the secondary IH cohort, five of seven children had headache and two of these improved (one after shunt revision and the other after angioplasty), whereas two lacked follow-up. Papilledema improved in one of three while the other two remained undocumented. Two IIH patients treated with angioplasty and one non-IIH child managed medically required ventriculoperitoneal shunting, whereas no stented patient in either subgroup did.

Regarding significant pressure gradients, four children had an initial venogram TSG > 10 mmHg (mean 21.6 ± 9.8 mmHg). Patients 7 and 12 underwent TSG during the initial venogram whereas Patient 9, whose initial gradient measured 11.8 mmHg, returned one month later with a repeat gradient of 23.5 mmHg; a stent was then implanted in that second session. Averaged across the three stented children, the gradient fell from 21 mmHg to 2.5 mm Hg, an 88% reduction, and every post-stent value lay below the 8-mmHg threshold regarded as hemodynamically significant. Headache resolved or markedly improved in all three children, papilledema resolved in two and improved in the third, and documented visual deficits normalized. None required ventriculoperitoneal shunting. The fourth patient (Patient 13, 35 mmHg) was managed conservatively after revision of a malfunctioning shunt normalized intracranial pressure, and no follow-up venography was obtained.

Five children were younger than twelve years at their first catheter session (mean 6.3 ± 3.7 years). Two of these younger patients, aged six and ten years, underwent unilateral stenting and achieved durable gradient normalization along with sustained resolution of presenting symptoms. Neither experienced a peri-procedural complication or required later CSF diversion. The remaining three patients, aged one, four and six years, were treated with balloon angioplasty alone. All tolerated the procedure without complications, but two later required VPS for persistent intracranial hypertension and the remaining patient required a shunt revision.

There were no perioperative complications (less than 30 days) in 19 of the 20 procedures. Patient 12 developed a retroperitoneal hemorrhage after deep circumflex iliac artery wire perforation during micro-puncture access for VSS. The child was managed non-operatively, with transfusion of packed red blood cells and temporary cessation of enoxaparin and recovered without sequelae. There were no deaths or permanent neurological deficits. No episodes of stent thrombosis, venous rupture, or intracranial hemorrhage were recorded. All children who underwent VSS tolerated dual antiplatelet therapy without clinically significant bleeding. Long-term (after 30 days), Patient 6 required a second stenting procedure due to the development of stenosis in a segment cranial to the initial stent.

## Discussion

Cerebral venous sinus stenosis (CVSS) remains rare in pediatric patients. It is often associated with idiopathic intracranial hypertension (IIH) and discovered during the work-up of IIH. Venous sinus pressures have been studied in adults with CVSS, yet in pediatric patients there is currently limited data [1, 9]. As one part of this study, we recorded the cerebral venous pressures associated with CVSS and location along the cerebral venous sinus where stenosis occurs.

Currently, most pediatric studies measuring venous sinus pressures focus on patients with IIH. Interestingly, in prepubertal patients with IIH, CVSS is rare. In a study by Riedel et al., one out of eleven prepubertal patients (average age 2.3 years) had CVSS, while the other 10 had normal intracranial anatomy [1]. Superior sagittal sinus pressures (SSSP) in Riedel et al.’s study averaged 18.9 mmHg with a daytime ICP of 12.9 mmHg and a nighttime ICP of 17.2 mmHg. In our cohort (15 patients with a median age of 13.0 years), every patient was diagnosed with CVSS. The average SSSP was 24.3 mmHg (SD 11.5; range 13–50 mmHg, *n* = 10) and mean CSF opening pressure was 409 mmH₂O. Eight of the fifteen children (53%) satisfied the revised Friedman criteria for IIH. Stenosis occurred primarily at the transverse sinus (26.7%) or transverse sinus/sigmoid sinus junction (53.3%). The trans-stenotic gradient (TSG) of 8.5 mmHg (IQR 4.0–13.3, n = 14) aligns closely with other studies in pediatric patients with CVSS (10.6—15.8 mmHg) [6, 8].

Patients with CVSS who are asymptomatic without evidence of elevated ICPs are generally observed without further intervention. Patients with CVSS and evidence of elevated ICPs require treatment. First line therapy is medical, predominantly with acetazolamide, but the best intervention after medical management remains under investigation. A recent large meta-analysis (1,066 patients, predominantly adults) demonstrates that cerebral venous sinus stenting (VSS) leads to a reduction in trans-stenotic gradient pressure and a significantly lower cerebrospinal fluid (CSF) opening pressure with associated improvements in tinnitus (95%), papilledema (89%), visual disturbances (88%), and headache (79%) [10]. For adults, the threshold for VSS is generally a TSG of ≥ 8 mmHg [7]. In patients who are unable to tolerate dual anti-platelet therapy or carry other contraindications to stenting, venous sinus angioplasty may provide short-term marginal improvement in IIH symptoms [11].

For pediatric patients, the threshold to intervene with VSS or venous angioplasty remains less clear. Schwarz et. al. used the TSG threshold of > 6 mmHg under general anesthesia and noted that the one pediatric patient who did not have a response from VSS was a patient who had a TSG of 7 mmHg pre-stenting. Of note, this patient did not improve after subsequent ventriculoperitoneal shunt placement and repeat suboccipital craniectomy with duraplasty either—suggesting patients with low TSGs may have an alternative pathophysiology for their IIH symptomology not related to CVSS [12].

**Fig. 1:**
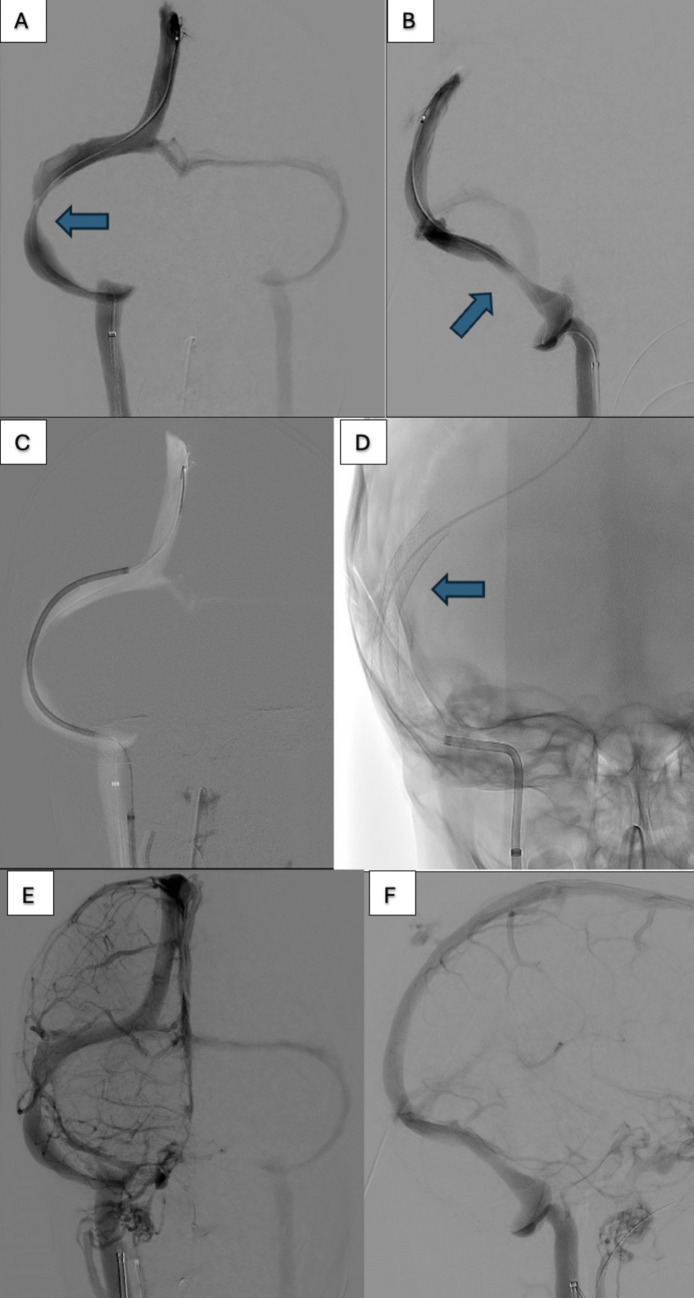
Case illustration of patient #7 demonstrating cerebral venous sinus stenosis treated with cerebral venous sinus stenting. Anterior–posterior **A** and lateral **B** views of pre-stent venogram showing cerebral venous sinus stenosis. Stent placement **C** followed by post-stent radiograph **D**. Venous phase of post-stenting angiogram on anterior–posterior **E** and lateral **F** views. Pressure gradient pre-stenting: 17.7 mmHg; post-stenting: 5.9 mmHg

In our study, five patients underwent first-time dural venous sinus stenting (mean pre-stent TSG of 17.0), with a significant average reduction in the trans-stenotic gradient of 13.5 mmHg (p = 0.04; 79.4% relative reduction). These included reductions from 11 to 2 mmHg, 11 to 8 mmHg, 17.7 to 5.9 mmHg, 23.5 to 1.5, 22 to 0 mmHg (Fig. 1). Sixty percent of patients had improvement of their papilledema, and 100% had headache resolution. Fifty percent of stented patients had reductions in their acetazolamide dosing. The median paired reduction in acetazolamide for the first time VSS group was −250 mg/day (p = 0.14) (Table 6).

VSS shows promise in treating pediatric IIH. Carter et al. (n = 12) noted that large decreases in TSG after stent placement in pediatric patients correlated with headache resolution (P = 0.0005) [13]. Another study focusing on pediatric patients with IIH post-VSS (n = 14) found that 60% of patients had reduced medication dosing, 85.7% had improved headaches, 100% had tinnitus resolution, and 80% had improvement of papilledema on follow-up ophthalmological examinations [8]. A systemic review on pediatric IIH reported that 82% of pediatric patients post-VSS had improved headaches and visual outcomes [6].

In our cohort treated with VSS (median follow-up 6 months), one out of five patients (20%) required further surgical intervention. This patient required a second venous stent due stent adjacent stenosis (SAS) cranial to where the initial stent was placed. SAS is known to occur in both adults and children. Other pediatric studies have found that it may occur up to 14–25% of the time—generally higher than what is seen in the adult population [8, 12, 14]. Additionally, treatment failure after VSS in adults is around 8.35%, while in pediatric patients it may be as high as 29% [8, 10, 12]. Higher rates of SAS and treatment failure in these pediatric studies is likely due to low powered studies, and these findings should be followed closely as further data is gathered to determine whether pediatric patients experience less long-term benefit from VSS than adults do.

Currently, there is no substantial literature regarding venous sinus angioplasty in pediatric patients. Three patients underwent venous angioplasty without complications. Yet, all three patients required further intervention. Patient 8, who had a ventriculoperitoneal shunt prior to angioplasty, required a shunt revision after the angioplasty due to uncontrolled ICPs and shunt failure. Patient 11 required placement of a ventriculoperitoneal shunt after angioplasty due to recurrence of symptoms and elevated ICPs. Patient 14 ultimately underwent ventriculoperitoneal shunt placement after undergoing multiple angioplasties without significant improvement of ICPs and papilledema.

Regarding peri-operative complications (within 30 days), one peri-operative (5%) complication out of 20 endovascular procedures occurred (procedures included DCV, diagnostic cerebral angiogram, VSS, and venous angioplasty). The procedure was complicated by a retroperitoneal hematoma following venous sinus stenting due to deep circumflex iliac artery perforation during femoral access, requiring close monitoring and supportive care initially, but without any deficits or long-term sequelae. In adults, complication rates for diagnostic cerebral venograms and VSS are around 5.4% [10]. In pediatric patients, serious complication rates remain low, including for VSS, and most perioperative complications are related to the access site (e.g. groin hematomas) [8, 12, 13].

The results of this study must be taken in the context of its limitations. A major limitation of this study is its small sample size, limiting the power of the study. Additionally, as a single center study based in the southeastern United States, it may not be generalizable to different institutions where the population of patients may be different along with the endovascular techniques performed. Expanding the study to a multicenter study in the future would help increase the power of the study. As a retrospective study, certain variables are not consistently collected across all patients or specific post-operative details may not be consistently recorded.

## Conclusion

Cerebral venous sinus stenosis (CVSS) remains rare within the pediatric population. In this study, the mean superior sagittal sinus pressure (SSSP) was 24.3 mmHg and the average trans-stenotic gradient (TSG) of all patients was 8.5 mmHg. Five patients underwent first time dural venous sinus stenting (mean pre-stent TSG of 17.0) with a significant reduction in the trans-stenotic gradient of 13.5 mmHg (p = 0.04; 79.4% relative reduction). All patients who were initially treated with VSS had a TSG ≥ 11 or more with 100% resolution of headaches and improvement of papilledema in 60% of patients. Peri-operative complications were 5%. Long-term, stent adjacent stenosis (SAS) occurred in 20% of VSS patients, supporting that SAS is an issue not only for adults, but pediatric patients as well. Of the patients who underwent venous sinus angioplasty, 100% required further surgical intervention for management of their intracranial pressures.

## Data Availability

No datasets were generated or analysed during the current study.

## References

[1] Riedel CS et al (2024) Elevated systemic venous pressures as a possible pathology in prepubertal pediatric idiopathic intracranial hypertension. Childs Nerv Syst 40(12):4203–4209 39254866 10.1007/s00381-024-06594-3 PMC11579111

[2] Matthews YY et al (2017) Pseudotumor cerebri syndrome in childhood: incidence, clinical profile and risk factors in a national prospective population-based cohort study. Arch Dis Child 102(8):715–721 28356250 10.1136/archdischild-2016-312238

[3] Bursztyn LL et al (2014) Has rising pediatric obesity increased the incidence of idiopathic intracranial hypertension in children? Can J Ophthalmol 49(1):87–91 24513363 10.1016/j.jcjo.2013.09.015

[4] Rangwala LM, Liu GT (2007) Pediatric idiopathic intracranial hypertension. Surv Ophthalmol 52(6):597–617 18029269 10.1016/j.survophthal.2007.08.018

[5] Albakr A et al (2016) Idiopathic intracranial hypertension in children: Diagnostic and management approach. Sudan J Paediatr 16(2):67–76 28096561 PMC5237838

[6] Friso S et al (2024) A systematic review of surgical and interventional radiology procedures for pediatric idiopathic intracranial hypertension. Front Pediatr 12:1466688 39539766 10.3389/fped.2024.1466688 PMC11557315

[7] Fargen KM et al (2018) Recommendations for the selection and treatment of patients with idiopathic intracranial hypertension for venous sinus stenting. J Neurointerv Surg 10(12):1203–1208 30030306 10.1136/neurintsurg-2018-014042

[8] Lee KE et al (2021) Dural venous sinus stenting for treatment of pediatric idiopathic intracranial hypertension. J Neurointerv Surg 13(5):465–470 32732257 10.1136/neurintsurg-2020-016183

[9] Lee K et al (2021) Correlation between intracranial pressure and venous sinus pressures in patients undergoing cerebral venography and manometry. J Neurointerv Surg 13(12):1162–1166 33674395 10.1136/neurintsurg-2020-017161

[10] Azzam AY et al (2024) Venous sinus stenting for idiopathic intracranial hypertension: an updated meta-analysis. J Neurol Sci 459:122948 38457956 10.1016/j.jns.2024.122948

[11] Carlos Martinez-Gutierrez J et al (2023) Primary balloon angioplasty of venous sinus stenosis in idiopathic intracranial hypertension. Interv Neuroradiol 29(4):358–362 35323053 10.1177/15910199221089446 PMC10399507

[12] Schwarz J et al (2021) Management of idiopathic intracranial hypertension in children utilizing venous sinus stenting. Interv Neuroradiol 27(2):257–265 33236688 10.1177/1591019920976234 PMC8050535

[13] Carter LM et al (2021) Venous sinus stenosis treatment in pediatric idiopathic intracranial hypertension: illustrative case and literature review. World Neurosurg 149:2–7 33476783 10.1016/j.wneu.2021.01.029

[14] Saber H et al (2018) Stent survival and stent-adjacent stenosis rates following venous sinus stenting for idiopathic intracranial hypertension: a systematic review and meta-analysis. Interv Neurol 7(6):490–500 30410529 10.1159/000490578 PMC6216784

